# Tetramethylpyrazine Enhances the Antitumor Effect of Paclitaxel by Inhibiting Angiogenesis and Inducing Apoptosis

**DOI:** 10.3389/fphar.2019.00707

**Published:** 2019-06-25

**Authors:** Liang Zou, Xiaowei Liu, Jingjing Li, Wei Li, Lele Zhang, Jian Li, Jinming Zhang

**Affiliations:** ^1^School of Medicine, Chengdu University, Chengdu, China; ^2^Key Laboratory of Coarse Cereal Processing, Ministry of Agriculture and Rural Affairs, Chengdu University, Chengdu, China; ^3^Department of Pharmacy and Pharmacology, Li Ka Shing Faculty of Medicine, The University of Hong Kong, Hong Kong, Hong Kong; ^4^School of Pharmacy, Chengdu University of Traditional Chinese Medicine, Chengdu, China

**Keywords:** tetramethylpyrazine, paclitaxel, angiogenesis, apoptosis, antitumor effect

## Abstract

Recent published findings have demonstrated the effectiveness of combining molecules from traditional Chinese medicine with chemotherapeutic drugs to treat cancer. Combined administration of these agents can overcome drug-mitigating responses as well as reduce adverse side effects, thereby enhancing the efficacy of the therapy. Tetramethylpyrazine (TMP), an alkaloid monomer from the medicinal herb *Ligusticum chuanxiong* hort, is known to exert a variety of antitumor effects including inhibition of tumor cell proliferation, metastasis, and drug resistance. In this research, we investigated antitumor effects of TMP combined with paclitaxel (PTX), a frontline chemotherapeutic drug, *in vitro* and *in vivo*. Our results indicate that TMP enhances the antitumor effects of PTX in ovarian cancer A2780 and SKOV3 cells. Furthermore, we found that combined treatment of TMP and PTX suppressed angiogenesis by inhibiting both ERK1/2 and Akt pathways and promoted apoptosis of tumor cells compared to TMP or PTX treatment alone. Moreover, TMP augmented the antitumor effects of PTX in ovarian cancer A2780 xenograft mouse models by significantly decreasing tumor burden and partially decreasing the toxicity of PTX, as evidenced by the decreased expression of proliferation and angiogenesis markers as well as the hematoxylin and eosin (H&E) staining and biochemical indexes assay. Overall, our findings provide novel mechanistic insight into the efficacy of combining of potent molecules present in traditional Chinese medicine with chemotherapeutic drugs for therapeutic intervention in cancer.

## Introduction

Cancer continues to pose a significant threat to human health worldwide (Siegel et al., 2017). For decades, chemotherapy has remained one of the most common and effective strategies for treating cancer. Drug-mitigating responses and adverse side effects are widely reported limitations of current chemotherapy treatments (Chidambaram et al., 2011; Nurgali et al., 2018). Paclitaxel (PTX) is a microtubule-stabilizing agent that is approved for the treatment of ovarian, breast, and lung cancer, among others (Weaver, 2014). It has long been used as a first-line chemotherapeutic drug for several cancer types. However, a large number of studies have reported acquired drug resistance as well as adverse side effects, which significantly limit the clinical usage of PTX (Yusuf et al., 2003).

Recently, intensive studies combining molecules from traditional Chinese medicine with chemotherapeutic drugs have validated the effectiveness of this strategy in overcoming limitations faced by chemotherapeutic agents and in improving the efficacy of the chemotherapy (Huang et al., 2018; Wang et al., 2018b). Glycyrrhizin, for example, has been shown to prevent oxidative stress by restoring antioxidant enzyme levels to protect malformation and renal toxicity caused by cisplatin in mice (Arjumand and Sultana, 2011). Similarly, apigenin combined with gemcitabine increases antitumor activity through induction of apoptosis as well as suppression of Akt and NF-κB activity *in vitro* and *in vivo* (Lee et al., 2008).

Tetramethylpyrazine (TMP) is an alkaloid monomer isolated from *Ligusticum chuanxiong* hort (Chen et al., 2018). A series of studies have shown that TMP possesses a variety of antitumor effects including the inhibition of tumor cell proliferation, invasion, and drug resistance (Wang et al., 2010). Reports have demonstrated that TMP administration induces apoptosis and autophagy in hepatocellular carcinoma (Cao et al., 2015). Additionally, treatment of A549 lung adenocarcinoma cells with TMP results in a significant inhibition of invasion, as well as suppression of A549 metastasis and COX-2 expression in a metastatic nude mouse xenograft model (Zheng et al., 2012). TMP also reverses multidrug resistance in breast cancer cells by regulating the expression and function of P-glycoprotein (Zhang et al., 2012).

In this study, combined administration of TMP with PTX was investigated *in vitro* and *in vivo* for antitumor effects. Furthermore, we sought to dissect the molecular mechanisms driving the antitumor effects of this combination. Our results indicate that TMP enhances the antitumor efficacy of PTX by inhibiting angiogenesis, inducing apoptosis, decreasing tumor burden in A2780 xenograft mouse models, and partially reducing the toxicity of PTX.

## Materials and Methods

### Materials

TMP (purity ≥99%) was purchased from Energy Chemical Co., Ltd (Shanghai, China); paclitaxel (PTX, purity ≥99%) was purchased from Dalian Meilun Biotech Co., Ltd (Dalian, China); vascular endothelial growth factor (VEGF) was bought from R&D systems (Minneapolis, MN, USA). TMP and PTX were dissolved in dimethyl sulfoxide (DMSO, Sigma-Aldrich Co., St. Louis, MO, USA) as stock solutions, and diluted in relative culture medium with final DMSO concentrations no higher than 0.1% upon using. VEGFR tyrosine kinase inhibitor II (VRI) was obtained from CalBiochem (San Diego, CA, USA). Antibodies recognizing phospho-p38 (Thr180/Tyr182), phospho-Akt (Ser 473), phospho-Erk1/2 (Thr202/Tyr204), Cleaved-Caspase 3, Cleaved-Caspase 9, Cleaved-PARP, and GAPDH were purchased from Cell Signaling Technology (Danvers, MA, USA). Antibodies against Ki67 and CD31 were purchased from Abcam (Cambridge, UK). Female BALB/c nude mice, aged 4–6 weeks (18–22 g), were obtained from Dashuo experimental animals Co., Ltd. (Chengdu, China) and maintained under specific pathogen-free conditions. All *in vivo* experiments including animal use and care protocols were carried out under the guidelines approved by the Institutional Animal Care and Use Committee of Chengdu University.

### Cell Culture

Human umbilical vein endothelial cells (HUVEC) were obtained from American Type Culture Collection (ATCC, USA) and cultured in F-12K complete media with 100 µg/ml heparin, 30 µg/ml endothelial cell growth supplement (ECGS), 10% heat-inactivated fetal bovine serum (FBS), and 1% penicillin–streptomycin (P/S). Cells at early passage (3−8 passages) were used for the outlined experiments. A2780 ovarian cancer cells were obtained from ATCC and maintained in DMEM culture medium, supplemented with 10% heat-inactivated FBS and 1% P/S. SKOV3 ovarian cancer cells were obtained from ATCC and cultured in McCoy’s 5A medium, supplemented with 10% heat-inactivated FBS and 1% P/S. All the cells were incubated at 37°C in a humidified atmosphere with 5% CO_2_.

### Cell Viability Assay

Cell viability was assessed using the MTT assay. Briefly, HUVEC cells, ovarian cancer A2780, and SKOV3 cells were seeded on 96-well plates at a density of 8 × 10^3^ cells/well. Cells were then treated with various concentrations of PTX (3.125−100 nM), TMP (6.25−200 µM), and the combination of the two compounds in low serum media (0.5%) for 48 h. Cells were treated with 0.5 mg/ml MTT. Four hours later, the formazan crystals were dissolved in 100 μl of DMSO and the absorbance was measured at 570 nm. Cell viability was calculated as the percentage of the controls. Each experiment was repeated at least three times.

### Cell Proliferation Assay

HUVEC cells were seeded into a 48-well plate at a density of 3 × 10^4^ cells per well in F-12K complete media and cultured for 24 h for cell attachment. Cells were then starved with low serum media (0.5% FBS) overnight to achieve a quiescent state. After starvation, cells were treated with 100 nM PTX, 100 µM TMP, or the combination in low serum media containing VEGF (50 ng/ml) for 48 h. Cell proliferation was detected by MTT assay.

### Transwell Migration Assay

The effects of TMP combined with PTX on the migration and invasion of HUVEC cells were examined using the transwell migration assays (8-μm pores). In the migration assay, the upper side of the membrane was pre-coated with collagen. HUVEC cells (5 × 10^4^ cells) were resuspended in 200 ml of low serum (0.5% FBS) medium containing 20 ng/ml VEGF + 100 nM PTX, 20 ng/ml VEGF + 100 µM TMP, 20 ng/ml VEGF + 100 nM PTX + 100 µM TMP. Cells were then deposited into the 24-well companion plate with 500 μl of low serum (0.5% FBS) medium containing the same concentration of agents as were used to coat the upper sides. Cells were incubated at 37°C for 24 h. Cells on the upper surface of the membrane were removed using cotton swabs. Inserts were fixed with 4% paraformaldehyde for 15 min, and then stained with 10 μg/ml of Hoechst 33342 for 15 min.

### Tube Formation Assay

Matrigel matrix (BD, Biosciences) was thawed at 4°C overnight and a 15-well u-slide was coated with 10 μl Matrigel/well and incubated at 37°C for 30 min for polymerization. HUVEC cells (1 × 10^4^ cells) were then resuspended in 50 μl of medium containing 0.5% FBS and 20 ng/ml VEGF + 100 nM PTX, 20 ng/ml VEGF + 100 µM TMP, 20 ng/ml VEGF + 100 nM PTX + 100 µM TMP, respectively, and seeded onto the Matrigel-coated plate. After 4-h incubation, HUVEC cells in the control group formed tube-like structures, which were defined as endothelial cord formations connected at both ends. Tube formation was quantified by counting the number of branching points in three randomly selected fields of view.

### Annexin V-FITC/PI Double Staining

An Annexin V-FITC assay kit was used to detect cell apoptosis. A2780 and SKOV3 cells were seeded at 2 × 10^5^ per well for 24 h and then incubated with 0.5% FBS medium containing 100 nM PTX, 100 µM TMP, or 100 nM PTX+100 μM TMP for 48 h. At the end of the incubation, cells were washed twice with cold phosphate-buffered saline (PBS), harvested, re-suspended in 200 μl of binding buffer, and stained with 3 μl of Annexin-FITC for 10 min, followed by 3 μl of PI solutions for 15 min. Cells were analyzed immediately by flow cytometry. Ten thousand events were counted for each sample.

### Mitochondrial Membrane Potential

A2780 and SKOV3 cells were seeded at 2 × 10^5^ per well for 24 h, then exposed to 100 nM PTX, 100 µM TMP, or 100 nM PTX + 100 μM TMP for 24-h incubation. After treatment, cells were collected and co-incubated with JC-1 dye (3 μg/ml) in incubation buffer for 20 min at 37°C. Cells were subsequently washed twice with PBS and examined by flow cytometry with 10,000 gated cells. The experiment was performed in triplicate.

### Western Blot Analysis

Protein was extracted using lysis buffer containing 1% phenylmethylsulfonyl fluoride and 1% protease inhibitor. Lysates were centrifuged at 12,500 × *g* for 20 min at 4°C, and the supernatant was collected. The total protein concentration was determined using a BCA Protein Assay kit. The lysates were subjected to Western blotting and incubated with phospho-Akt, phospho-Erk1/2, phospho-p38, Cleaved-PARP, Cleaved-Caspase 3, Cleaved-Caspase 9, and GAPDH antibodies. Images of protein bands were captured using a Molecular Imager ChemiDoc XRS (Bio-Rad Laboratories, Hercules, CA, USA). Densitometric measurements of band intensity were performed using Quantity One Software (Bio-Rad Laboratories, Hercules, CA, USA).

### *In Vivo* Antitumor Activity Study

Subcutaneous implantation of human cancer cells in nude mice represents one of the most common *in vivo* model with potential clinical relevance, and has been widely used for preclinical evaluation of anticancer drugs.* In vivo* antitumor studies were performed on A2780-heterografted female BALB/c nude mice. When tumor volumes reached approximately 200 mm^3^, the mice were divided into four groups (eight mice per group): control, PTX-treated, TMP-treated, and PTX+TMP-treated. All components were dissolved in polyoxyethylene castor oil and ethanol mixture (1:1, v:v) and were diluted with normal saline to required concentrations before administration. According to the previous references, 5 mg/kg of PTX and 60 mg/kg of TMP were used for the *in vivo* study, and they were administered by tail vein injection once every 2 days (Wang et al., 2014; Zhang et al., 2017). The tumor volumes were measured every other day in order to evaluate the antitumor activity. Additionally, the weights of the mice were recorded to illustrate the systematic toxicity. All of the mice were sacrificed after seven doses and the tumor tissue weights were recorded to calculate the tumor volumes using the formula: V = α·β^2^/2, in which α and β represent the length and width of the tumors, respectively. All nude mice were sacrificed on the 12th day and the tumor tissues, hearts, livers, spleens, lungs, and kidneys were harvested. After polyformaldehyde fixing, paraffin embedding, and 5-μm serial slicing, the tumors were dyed with hematoxylin and eosin (H&E) and were histologically analyzed (Cheng et al., 2017). Part of the tumor tissues were subjected to immunohistochemical staining as previously reported (Wang et al., 2018a). Liver tissues of the mice were also collected for biochemical analysis. Alanine transaminase (ALT), aspartate transaminase (AST), alkaline phosphatase (ALP), and albumin (ALB) levels were detected using the relative detection kits according to the manufacturer’s instructions.

### Statistical Analysis

All data are expressed as mean ± SD for at least three independent experiments. Data were analyzed by GraphPad Prism 5.0. Statistical significance was assessed by one-way analysis of variance, and a *p*-value less than 0.05 (*P* < 0.05) was considered to be significant.

## Results

### TMP Enhances the Antitumor Effect of PTX in Tumor Cells

**Figure 1:**
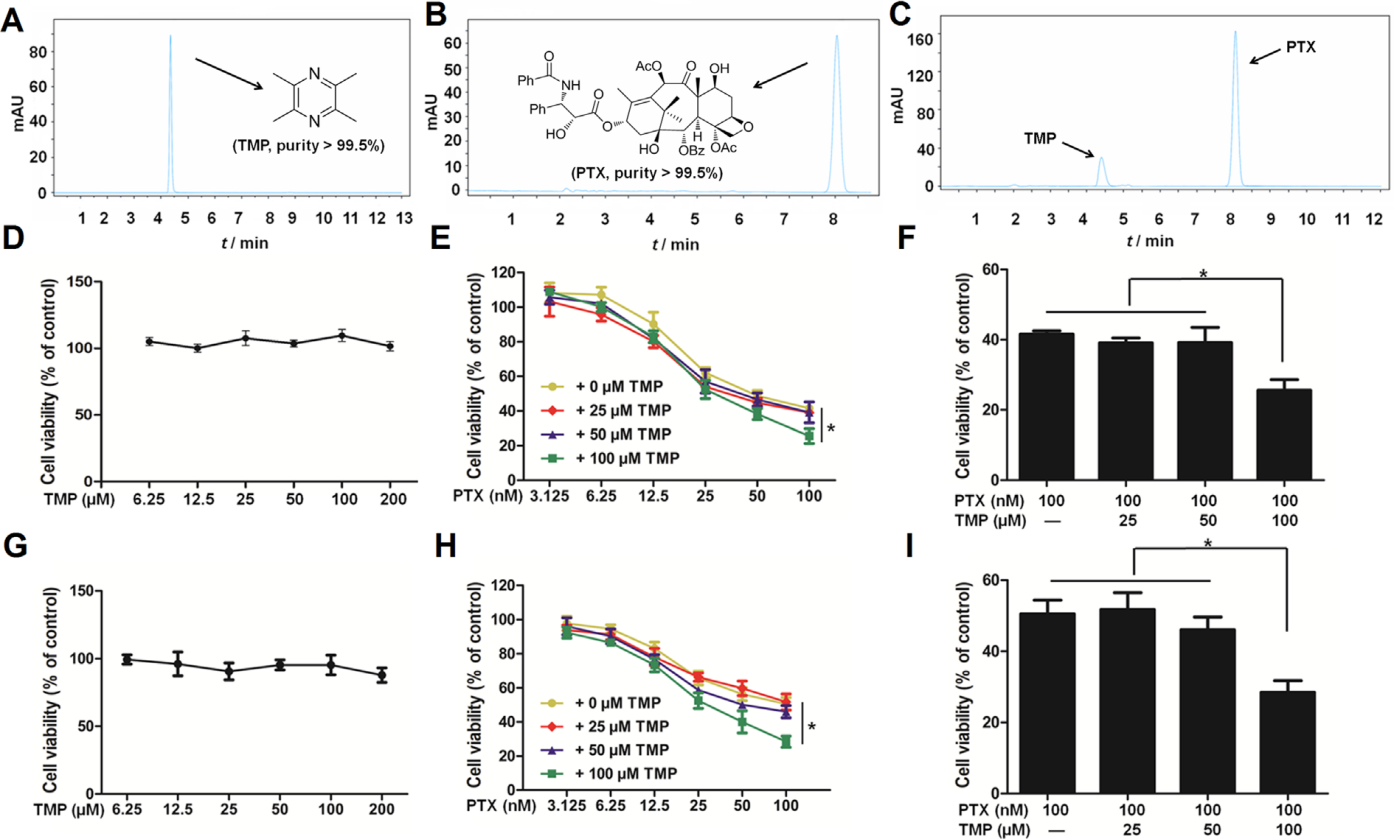
Tetramethylpyrazine (TMP) enhances the inhibitory effect of paclitaxel (PTX) in ovarian cancer A2780 cells. **(A, B)** Chromatogram and chemical structures of PTX and TMP. **(C)** Chromatogram of PTX and TMP mixture. **(D–F)** A2780 cells were treated with serial concentrations of PTX, TMP, or PTX+TMP for 48 h and cell viability was detected by MTT assay. **(G–I)** SKOV3 cells were treated with serial concentrations of PTX, TMP, or PTX+TMP for 48 h and cell viability was detected by MTT assay. Data are expressed as mean ± SD of three independent experiments (*P < 0.05).

Before performing *in vitro* cellular assays, we employed high pressure liquid chromatography with ultraviolet (HPLC-UV) methods to assess the purity of PTX and TMP as well as the potential interaction between them. We determined the purity of both compounds to be >99.5% (**Figure 1A, B**). Meanwhile, there was no detectable interaction between the two compounds (**Figure 1C**). We then tested the cytotoxicity of PTX, TMP, and the combination of the two compounds in ovarian cancer A2780 and SKOV3 cells. We treated A2780 cells with TMP, reaching the highest concentration of 200 μM, under which minimal to no inhibitory effect was observed (**Figure 1D**). We performed a serial fold dilution of PTX starting from 100 nM and found PTX exhibited an inhibitory effect at concentrations higher than 6.25 nM. When the concentration of PTX was raised to 100 nM, 48.8% of the A2780 cells survived (**Figure 1E**). We then combined either 25, 50, or 100 μM of TMP with up to 100 nM of PTX. The combinations of 100 nM PTX with 25 or 50 μM TMP generated cell survival percentages of 39.1% and 39.2%, respectively, which was comparable to PTX alone (41.6%) (**Figure 1E**). In contrast, the combination of 100 μM of TMP with 100 nM of PTX dramatically decreased the survival rate of A2780 cells to 25.6%, which was significantly different from the PTX alone group (**Figure 1F**). In SKOV3 cells, TMP exhibited no inhibitory effect at concentrations up to 200 μM (**Figure 1G**); 100 nM PTX generated cell survival percentages of 50.6%, while the combinations of 25 or 50 μM TMP didn’t improve the inhibitory effect of 100 nM PTX (**Figure 1H**). In contrast, the combination of 100 μM of TMP with 100 nM of PTX decreased viability of SKOV3 cells to 28.5% (**Figure 1I**).

### TMP Enhances the Inhibitory Effect of PTX in HUVEC Cells

**Figure 2:**
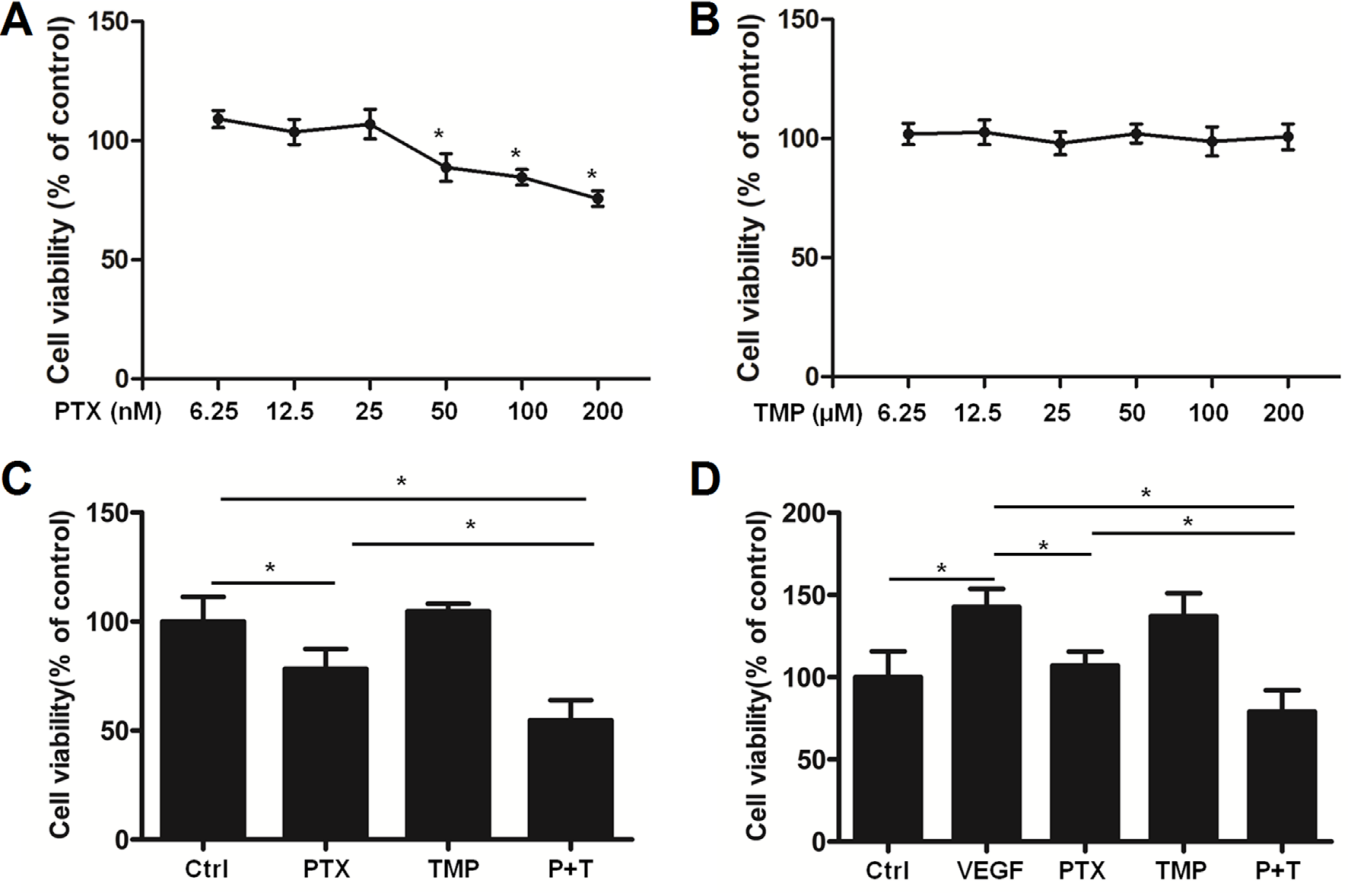
TMP enhances the inhibitory effect of PTX in HUVEC cells. **(A, B)** HUVEC cells were treated with serial concentrations of PTX or TMP for 48 h. Cell viability was detected by MTT assay. **(C)** HUVEC cells were treated with 100 nM PTX, 100 µM TMP, or PTX+TMP for 48 h, and cell viability was detected by MTT assay. **(D)** HUVEC cells were treated with 100 nM PTX, 100 µM TMP, or PTX+TMP in the presence of vascular endothelial growth factor (VEGF) for 48 h and cell viability was detected by MTT assay. Data are expressed as mean ± SD of three independent experiments (*P < 0.05).

We then used HUVEC cells as an *in vitro* angiogenesis model to analyze the effects of PTX, TMP, and the combination of the two compounds on VEGF-induced endothelial cell proliferation. A serial fold dilution of PTX was performed starting from 200 nM (**Figure 2A**). Concentrations higher than 25 nM exhibited an inhibitory effect on HUVEC cells, which became significantly different from control conditions at 50 nM and above (*P* < 0.05; **Figure 2A**). For TMP, the highest concentration tested was 200 μM, under which no dramatic inhibitory effect was observed (**Figure 2B**). PTX and TMP combined treatment decreased the survival rate of HUVEC cells compared to PTX alone (*P* < 0.05; **Figure 2C**), suggesting that TMP enhanced the inhibitory effect of PTX in proliferation of HUVEC cells. We then administered PTX and TMP alone and in combination to HUVEC cells in the presence of VEGF, using the VEGF-treated group alone as a positive control. HUVEC cells treated only with VEGF displayed an increase in viability of approximately 1.5 times that of control, while the combination significantly reduced HUVEC cell vitality approximately 0.75 times that of control. This decrease in viability was significant compared to PTX-treated cells alone (*P* < 0.05) (**Figure 2D**), suggesting that the combination group could overcome angiogenesis-promoting conditions by inhibiting cell proliferation.

### TMP Promotes the Anti-Angiogenic Effect of PTX

**Figure 3:**
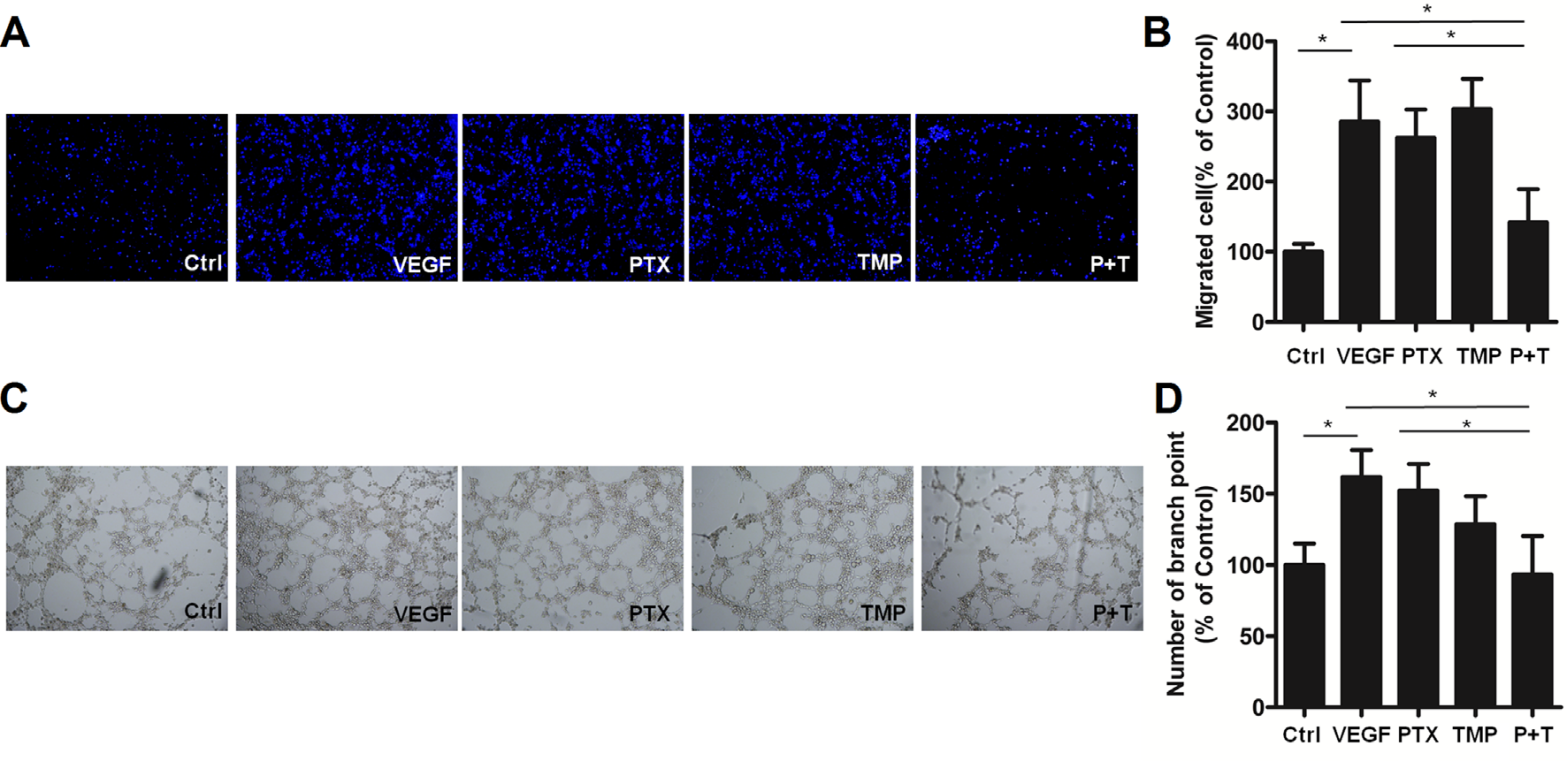
TMP enhances the inhibitory effect of PTX on migration and tube formation of HUVEC cells. **(A)** Migration of HUVEC cells was detected using transwell assays. Cells were treated with 100 nM PTX, 100 µM TMP, or PTX+TMP in the presence of VEGF for 48 h and then stained with Hoechst 33342. **(B)** Statistical analysis of transwell migration results. **(C)** HUVEC cells were treated with 100 nM PTX, 100 µM TMP, or PTX+TMP in the presence of VEGF for 48 h prior to detecting tube formation. **(D)** Tube formation was quantified by counting the number of branching points in three randomly selected fields of view. Images were captured under 20× magnification. Data are expressed as mean ± SD of three independent experiments (*P < 0.05).

In addition to proliferation, angiogenesis is also involved in metastasis and tube formation. For this reason, we investigated the anti-angiogenic effects of combining TMP with PTX by analyzing metastatic potential and tube formation of HUVEC cells. HUVEC cells were subjected to transwell migration assays and then stained with Hoechst 33342. Hoechst staining revealed similar cell numbers among TMP, PTX, and VEGF-treated groups, which was significantly higher than that of control (**Figure 3A**). Quantitative analysis by ImageJ software showed that PTX alone and VEGF were approximately three-folds higher than that of the control (**Figure 3B**). However, the combination of TMP and PTX significantly reduced the number of HUVEC cells to about half of the PTX group alone (*P* < 0.05), which indicates that the combination of TMP and PTX inhibits angiogenesis by reducing cell metastasis. On the other hand, tube formation assessment revealed that VEGF treatment increased the number of small tubes, while PTX and TMP decreased the number of small tubes. Moreover, co-treatment of PTX and TMP markedly decreased the number of small tubes (**Figure 3C, D**), indicating that the anti-angiogenic effect of PTX was dramatically enhanced by TMP.

**Figure 4:**
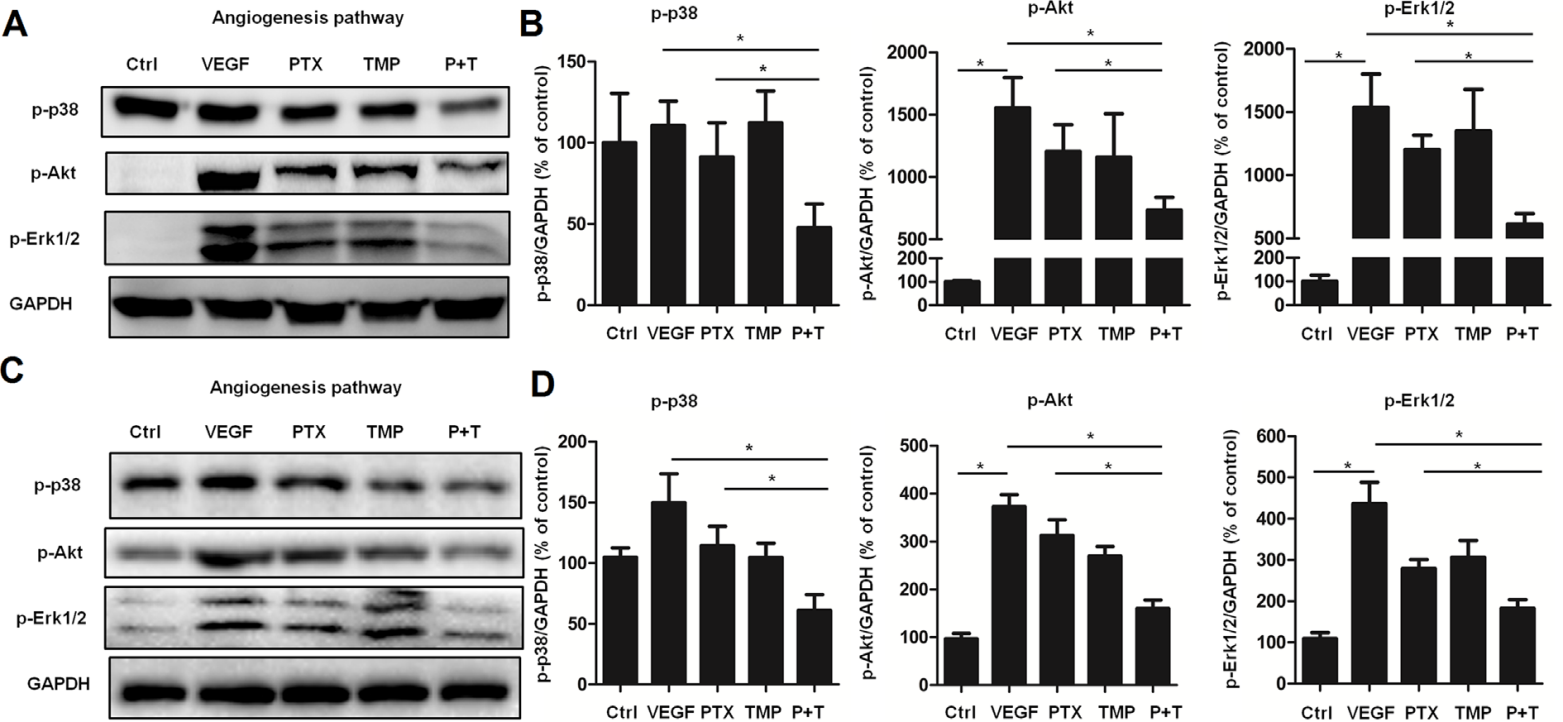
TMP combined with PTX suppresses the expression of proteins involved in angiogenesis. **(A, B)** A2780 cells were treated with 20 ng/ml VEGF or VEGF combined with 100 nM PTX, 100 µM TMP, PTX+TMP for 48 h. Western blot analysis was performed to evaluate the expression levels of p-p38, p-Akt, and p-Erk1/2, and representative blots are shown with densitometry. **(C, D)** HUVEC cells were treated with VEGF alone or VEGF combined with 100 nM PTX, 100 µM TMP, PTX+TMP for 48 h. Western blot analysis was performed to evaluate the expression levels of p-p38, p-Akt, and p-Erk1/2, and representative blots are shown with densitometry. Data are expressed as mean ± SD of three independent experiments (*P < 0.05).

To further explore the mechanism by which PTX combined with TMP inhibits angiogenesis, the p38, Akt, and Erk1/2 pathways were investigated by Western blot analysis. In ovarian cancer A2780 cells, TMP combined with PTX showed lower levels of p38 phosphorylation than VEGF or PTX alone (**Figure 4A, B**). The TMP+PTX combination reduced the phosphorylation of proliferative pathway mediators Erk1/2 and Akt (**Figure 4A, B**). In addition, TMP+PTX combination also decreased the phosphorylation of p38, Erk1/2, and Akt in HUVEC cells (**Figure 4C, D**). These results further suggest that TMP+PTX treatment attenuates angiogenesis by down-regulating the activity of proteins involved in driving angiogenesis.

### TMP Promotes Tumor Cell Apoptosis Induced by PTX

**Figure 5:**
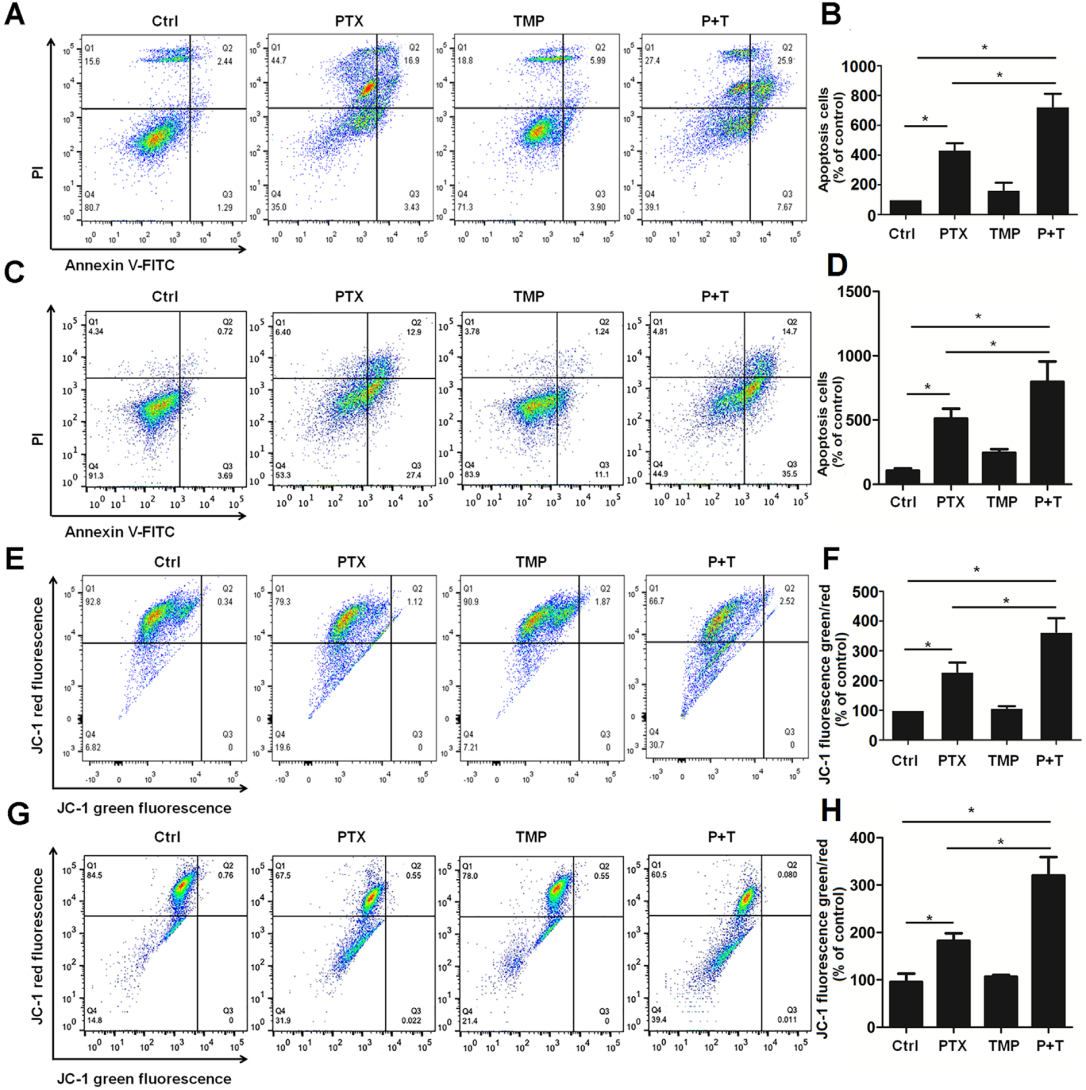
TMP promotes PTX-induced apoptosis and mitochondrial membrane potential loss in ovarian cancer A2780 and SKOV3 cells. **(A, B)** A2780 cells were treated with 100 nM PTX, 100 µM TMP, or PTX+TMP for 48 h. Cell apoptosis was detected by using Annexin V-FITC/PI double staining, and percentage of apoptosis cells were calculated. **(C, D)** A2780 cells were treated with 100 nM PTX, 100 µM TMP, or PTX+TMP for 24 h. Mitochondrial membrane potential of cells was analyzed by JC-1 staining, and JC-1 fluorescence ratios (green/red) were calculated. **(E, F)** SKOV3 cells were treated with 100 nM PTX, 100 µM TMP, or PTX+TMP for 48 h. Cell apoptosis was detected by using Annexin V-FITC/PI double staining, and percentage of apoptosis cells were calculated. **(G, H)** SKOV3 cells were treated with 100 nM PTX, 100 µM TMP, or PTX+TMP for 24 h. Mitochondrial membrane potential of cells was analyzed by JC-1 staining, and JC-1 fluorescence ratios (green/red) were calculated. Data are expressed as mean ± SD of three independent experiments (*P < 0.05).

TMP has been reported to promote tumor cell apoptosis (Cao et al., 2015). As a result, we also explored the antitumor effect as well as the mechanism of TMP combined with PTX as it relates to apoptosis. Annexin V-FITC/PI apoptosis detection was performed to compare control, PTX alone, TMP alone, and PTX+TMP groups by flow cytometry. In A2780 cells, the apoptotic cells in the PTX group were approximately three-folds higher than that of the control group, while the PTX+TMP group was approximately six-folds higher (**Figure 5A, B**). In SKOV3 cells, PTX treatment induced about four-folds higher percentage of apoptosis, while PTX+TMP induced approximately seven-folds higher percentage of apoptosis (**Figure 5C, D**). Thus, TMP co-treatment remarkably promoted apoptosis induced by PTX in ovarian cancer A2780 and SKOV3 cells.

Decreased mitochondrial membrane potential is an early event in apoptosis (Yang et al., 2014;Chatterjee et al., 2016). JC-1 can be used as a flow cytometry marker to detect the changes in mitochondrial membrane potential. Normal cells have higher mitochondrial membrane potential voltages to form JC-1 aggregates, which emit red fluorescence, while cells with reduced mitochondrial membrane potential that form JC-1 monomers will emit green fluorescence. As can be seen from the control groups in **Figure 5E–H**, most cells showed red fluorescence while only few percentage of the cells showed green fluorescence in both A2780 and SKOV3 cells. TMP treatment didn’t induce obvious change in JC-1 red fluorescence. Otherwise, compared with the PTX group, the ratio of JC-1 green fluorescence to JC-1 red fluorescence in the PTX+TMP group was significantly higher than that of control as well as TMP and PTX alone groups, suggesting that PTX+TMP treatment induces apoptosis by reducing mitochondrial membrane potential in both A2780 (**Figure 5E, F**) and SKOV3 (**Figure 5G, H**) cells.

**Figure 6:**
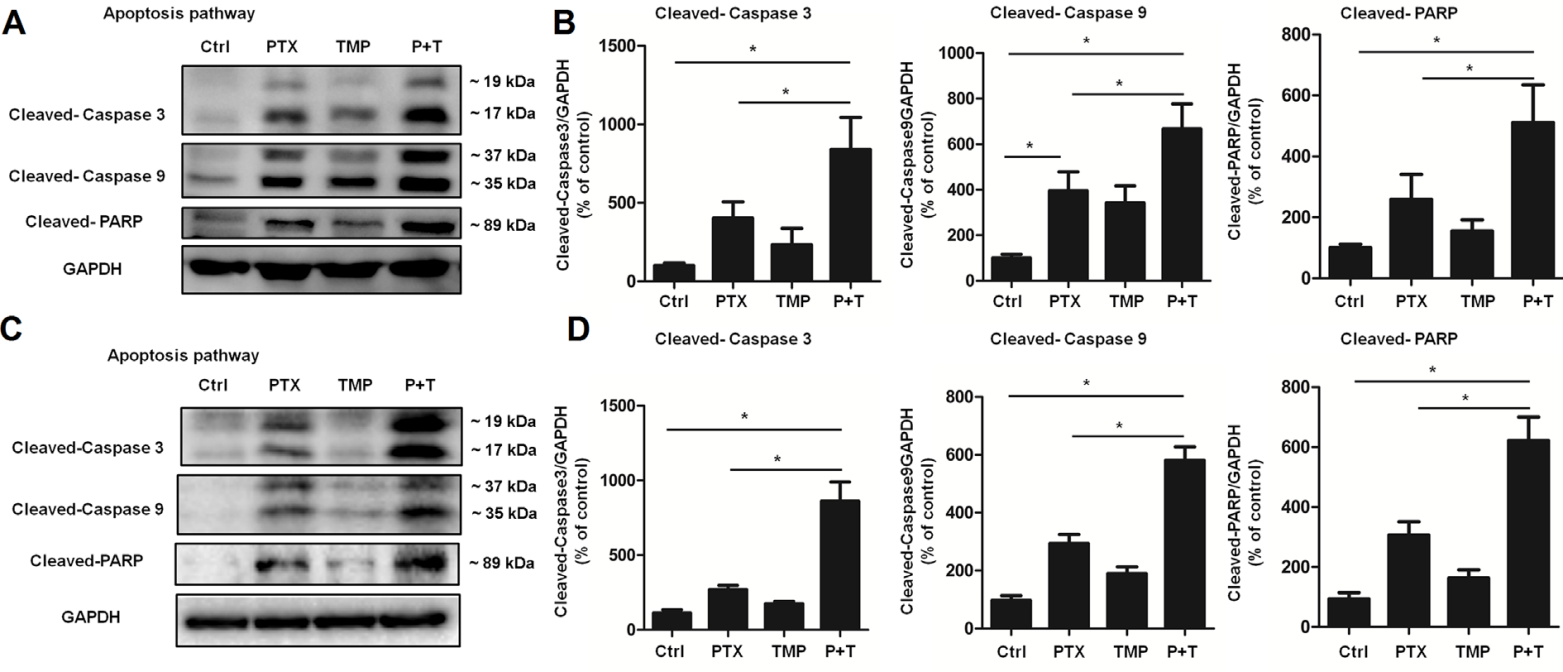
TMP enhances PTX-induced up-regulation of proteins involved in apoptosis. **(A, B)** A2780 cells were treated with 100 nM PTX, 100 µM TMP, or PTX+TMP for 48 h. Protein levels of Cleaved-Caspase 3, Cleaved-Caspase 9, and Cleaved-PARP were detected by Western blot analysis, and densitometry of the proteins were calculated. **(C, D)** SKOV3 cells were treated with 100 nM PTX, 100 µM TMP, or PTX+TMP for 48 h. Protein levels of Cleaved-Caspase 3, Cleaved-Caspase 9, and Cleaved-PARP were detected by Western blot analysis, and densitometry of the proteins was calculated. Data are expressed as mean ± SD of three independent experiments (*P < 0.05).

Next, we explored the mechanism of PTX+TMP treatment in promoting apoptosis from the molecular level. Specifically, we investigated the expression of apoptotic proteins caspase-3, caspase-9, and their downstream factor PARP. The results showed that PTX treatment partially activated caspase-3, caspase-9, and PARP in A2780 (**Figure 6A, B**) and SKOV3 (**Figure 6C, D**) cells. When TMP was combined with PTX, TMP significantly enhanced the pro-apoptotic ability of PTX (*P* < 0.05), which strongly suggests that TMP can enhance the antitumor effect of PTX by promoting apoptosis.

### TMP Enhances the Antitumor Effect of PTX *In Vivo*

**Figure 7:**
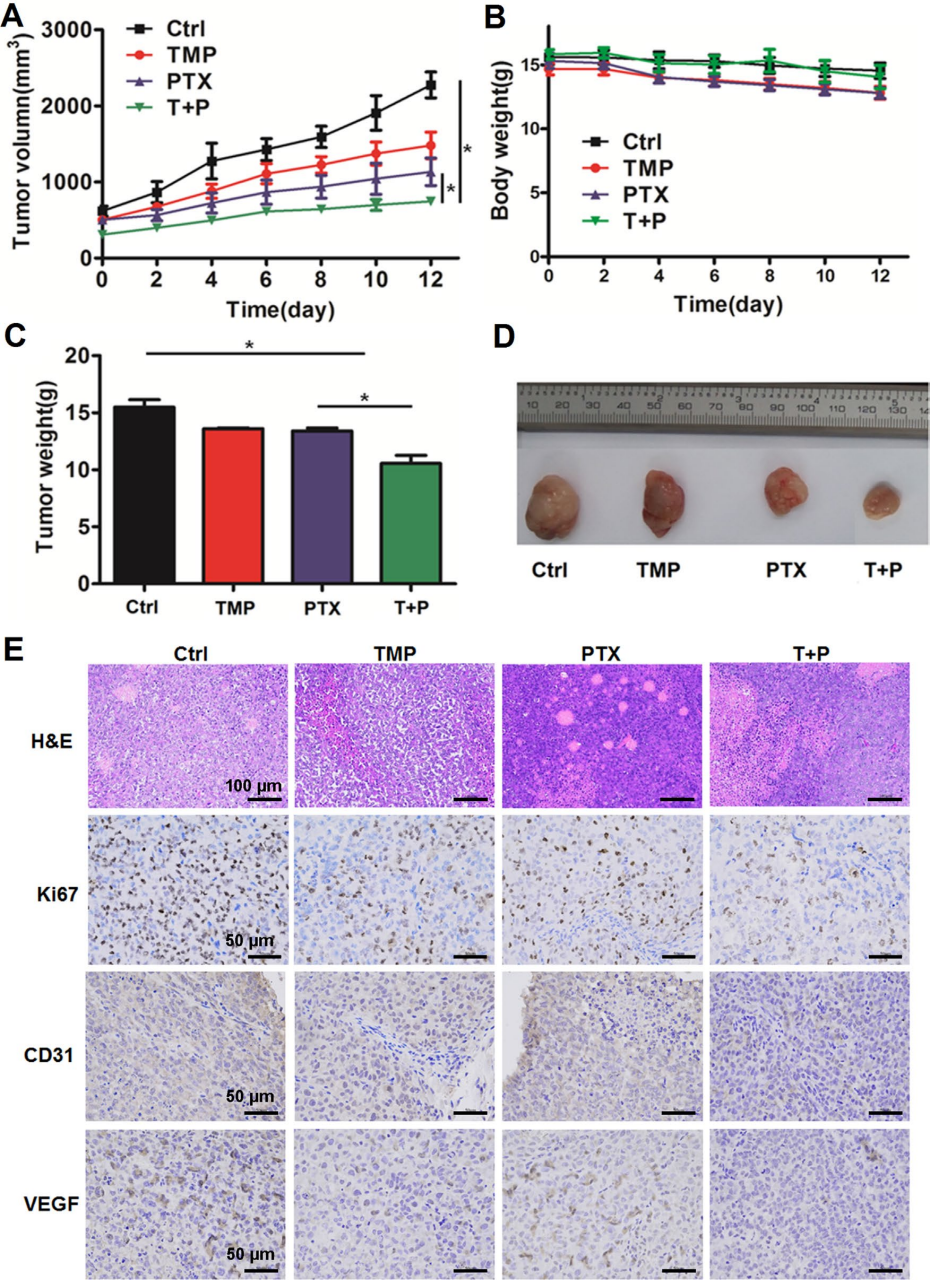
TMP enhances the antitumor effect of PTX *in vivo*. **(A)** Average tumor volumes of control (Ctrl), TMP (60 mg/kg), PTX (5 mg/kg), and TMP+PTX groups. **(B)** Recorded body weight of the mice. **(C, D)** Tumor weight measurements and size depictions of tumors from each group. **(E)** The tumors were subjected to hematoxylin and eosin (H&E) staining. The expression of Ki67, CD31, and VEGF was determined by immunohistochemical staining of tumors from the four groups. Data are expressed as mean ± SD of three independent experiments (*P < 0.05).

A2780 xenograft mouse models were used to study the antitumor efficacy of PTX+TMP combined treatment in ovarian cancer. After administrating the combination treatment to mice for 12 days, the tumors in the control group reached 2,000 mm^3^ in volume, while tumors in the PTX group grew to a maximum of 1,000 mm^3^ (**Figure 7A**). The tumor volume in the combined treatment group was just over 500 mm^3^, indicating that the combined treatment significantly reduced the growth rate of the tumor (**Figure 7A**). In addition, combined administration of PTX and TMP did not significantly reduce the body weight of the mice (**Figure 7B**). Upon reaching experimental endpoint, tumors were harvested, weighed, and compared for overall size, indicating that PTX and TMP co-treatment further decreased the tumor weight and tumor size compared with PTX alone (**Figure 7C, D**).

H&E staining was performed to evaluate the histological changes in the tumors. As shown in **Figure 7E**, tumors from all groups displayed irregular shapes, contained large nuclei with nuclear heterogeneity, noticeable pathological mitosis, and significantly enlarged nuclear/cytoplasmic ratio complicated with vascular proliferation primarily located in the interstitium or on the tumor border. Tissue necrosis zones could also be observed in each group. Varying levels of cancer cell degeneration occurred in the TMP group, the PTX group, and the TMP+PTX group, which was the most severe. Interestingly, clear decreases in the vascular component were noticeable in the TMP group and the TMP+PTX group. Meanwhile, immunohistochemical staining also indicated that TMP co-treatment further decreased the expression of proliferation and angiogenesis markers including Ki67, CD31, and VEGF compared with the TMP alone and PTX alone groups (**Figure 7E**).

**Figure 8:**
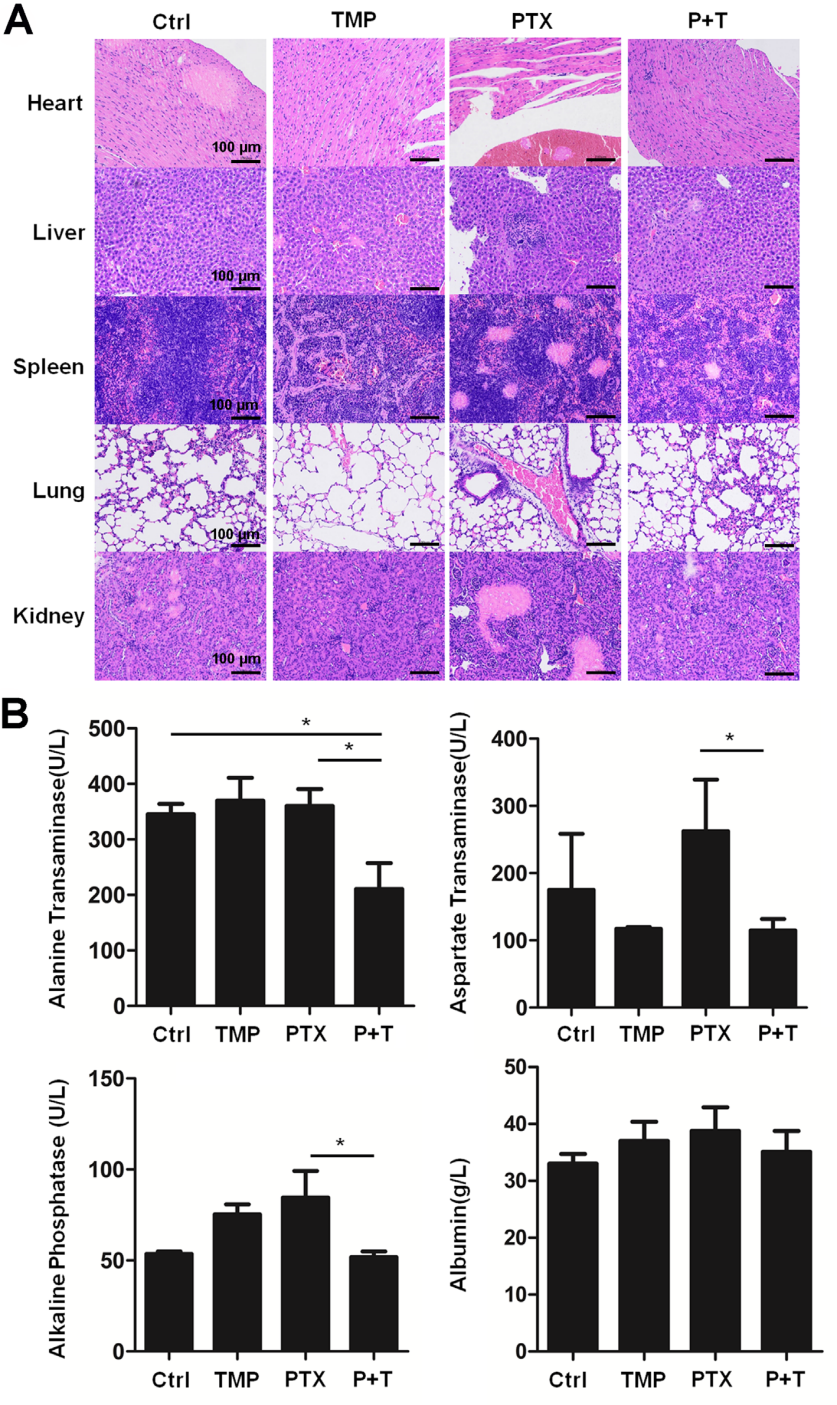
TMP partially decreased the toxicity of PTX *in vivo*. **(A)** Histologic assessments of major organs with H&E staining. **(B)** Alanine transaminase, aspartate transaminase, alkaline phosphatase, and albumin levels in liver were measured. Data are expressed as mean ± SD of three independent experiments (*P < 0.05).

Moreover, H&E staining in the tissue sections of the heart, liver, spleen, lung, and kidney indicates the ability of TMP to partially decrease the tissue damage induced by PTX (**Figure 8A**). To further confirm the effect, ALT, AST, ALP, and ALB levels in the liver of the mice were detected. Specially, we found TMP co-treatment suppressed the levels of ALT, AST, and ALP in the liver tissues (**Figure 8B**). These observations suggested that TMP partially decreased the toxicity of PTX.

## Discussion

For decades, the advancements in targeted cancer therapy and immunotherapy have considerably improved the clinical treatment of patients suffering from various types of cancer (Pardoll, 2012;Chan and Hughes, 2015). However, these modern therapies also experience intractable issues, such as acquired resistance and low tumor response rates, among other issues (Camidge et al., 2014; Sharma et al., 2017). Chemotherapy continues to play a very important role in the treatment of cancer, especially for the patients who do not respond to or experience adverse effects of targeted therapy and immunotherapy. Considering the widely reported limitations of chemotherapy, combining ingredients from traditional Chinese medicine with chemotherapeutic drugs has received heightened attention. A number of compounds from Chinese medicine have been reported to improve the therapeutic outcomes of cancer patients when used in combination with chemotherapeutic drugs. Combining these treatments can induce synergetic effects, inhibit side effects, or overcome drug resistance (Huang et al., 2018). In the current study, we found that the combination TMP with PTX resulted in remarkable enhancement of the pro-apoptotic effects of PTX.

In the previous study, we have screened a number of concentrations for TMP and PTX. The concentrations used *in vitro* were selected according to the results of the preliminary experiments. Take A2780 cells as an example; the cell survival percentage remains about 40–50% even when the concentration of PTX raised up to 2 μM. Thus, we selected 100 nM PTX for further study as it already generated cell survival percentage of about 50%. For TMP, we also screened serial concentrations up to 200 μM. However, we found that although TMP exhibited no inhibitory effect even at a concentration of 200 μM, it could partially increase the inhibitory effect of PTX at 100 μM. Since 100 nM PTX has already decreased viability of cells to about 50%, 100 μM could further promote the inhibitory rate for more than 20%. The effects and mechanisms are worth further investigation.

Previous work has demonstrated that angiogenesis is one of the most important targets in antitumor treatment (Fukumura and Jain, 2007). Furthermore, PTX has been demonstrated to possess anti-angiogenic activity as a promoter of microtubule polymerization (Lau et al., 1999). In our previous study, we found that TMP may interact with VEGFR2 and inhibit VEGF signaling (data not shown). Thus, we further investigated whether the combination of TMP and PTX could impart anti-angiogenic effects in the current study. Our results indicate that TMP promotes the anti-angiogenic effect of PTX, which may be at least partially mediated by the inhibition of ERK1/2 and Akt pathways. Besides, it has been generally accepted that mitochondria potential change is closely related with cell apoptosis (Desagher and Martinou, 2000). Upon apoptotic stimulation, cytochrome c released from the mitochondria may associate with Caspase 9, and the following intrinsic proteolytic processing promotes the cleavage of Caspase 9; the cleaved-Caspase 9 further activates other caspase members to initiate a caspase cascade and leads to cell apoptosis (Bratton and Salvesen, 2010). Our results suggest that the combination of TMP and PTX can activate caspase 9 and induce mitochondrial membrane potential loss, indicating that the mitochondria mediated apoptosis pathway is involved during this process. Thus, both anti-angiogenic effects and mitochondria mediated apoptosis likely contribute to the antitumor activity of the combination treatment.

In addition, we further investigated the antitumor effect of TMP and PTX *in vivo*. We found that TMP enhances the antitumor effect of PTX and also partially alleviates the toxicity of PTX. However, we recognize that, due to different pharmacokinetic properties, different drugs may not arrive at the targeted lesion simultaneously despite concurrent administration. Therefore, appropriate pharmacokinetic studies should be performed to further improve the efficacy of the combination therapy. Based on the potent effects of combining PTX and TMP, preparing prodrugs by modifying their chemical structures may enhance the targeting effect in optimized tumor treatments.

In conclusion, our study demonstrates that TMP promotes the antitumor effect of PTX. TMP combined with PTX suppresses angiogenesis by inhibition of the ERK1/2 and Akt pathways and promotes apoptosis of tumor cells. Moreover, TMP enhances the antitumor effect of PTX *in vivo* and also partially alleviates the toxicity of PTX. Given our results, TMP should be further developed as a potential anticancer candidate due to its enhancement of the antitumor effect of PTX.

## Ethics Statement

All *in vivo* experiments including animal use and care protocols were carried out under the guidelines approved by the Institutional Animal Care and Use Committee of Chengdu University.

## Author Contributions

LZ and JZ conceived and designed the research work. LZ, XL, JL, and WL performed the experiments. LZ and JL provided professional assistance in manuscript drafting. LZ and XL wrote the manuscript.

## Funding

This research was supported by the Project of Sichuan Provincial Education Department (17TD0010), Project of Sichuan Provincial Science and Technology Department (2019YJ0661), and Young Elite Scientiests Sponsorship Program by CAST (No. QNRC1-01).

## Conflict of Interest Statement

The authors declare that the research was conducted in the absence of any commercial or financial relationships that could be construed as a potential conflict of interest.
